# Spatial distribution of people diagnosed with tuberculosis through routine and active case finding: a community-based study in Kampala, Uganda

**DOI:** 10.1186/s40249-020-00687-2

**Published:** 2020-06-22

**Authors:** Katherine O. Robsky, Peter J. Kitonsa, James Mukiibi, Olga Nakasolya, David Isooba, Annet Nalutaaya, Phillip P. Salvatore, Emily A. Kendall, Achilles Katamba, David Dowdy

**Affiliations:** 1 Department of Epidemiology, Johns Hopkins Bloomberg School of Public Health, Baltimore, MD USA; 2 Uganda Tuberculosis Implementation Research Consortium, Makerere University, Kampala, Uganda; 3 Johns Hopkins School of Medicine, Baltimore, MD USA; 4 Department of Medicine, Clinical Epidemiology and Biostatistics Unit, Makerere University, College of Health Sciences, Kampala, Uganda

**Keywords:** Tuberculosis, Epidemiology, Health system, Geographic information systems

## Abstract

**Background:**

Routine tuberculosis (TB) notifications are geographically heterogeneous, but their utility in predicting the location of undiagnosed TB cases is unclear. We aimed to identify small-scale geographic areas with high TB notification rates based on routinely collected data and to evaluate whether these areas have a correspondingly high rate of undiagnosed prevalent TB.

**Methods:**

We used routinely collected data to identify geographic areas with high TB notification rates and evaluated the extent to which these areas correlated with the location of undiagnosed cases during a subsequent community-wide active case finding intervention in Kampala, Uganda. We first enrolled all adults who lived within 35 contiguous zones and were diagnosed through routine care at four local TB Diagnosis and Treatment Units. We calculated average monthly TB notification rates in each zone and defined geographic areas of “high risk” as zones that constituted the 20% of the population with highest notification rates. We compared the observed proportion of TB notifications among residents of these high-risk zones to the expected proportion, using simulated estimates based on population size and random variation alone. We then evaluated the extent to which these high-risk zones identified areas with high burdens of undiagnosed TB during a subsequent community-based active case finding campaign using a chi-square test.

**Results:**

We enrolled 45 adults diagnosed with TB through routine practices and who lived within the study area (estimated population of 49 527). Eighteen zones reported no TB cases in the 9-month period; among the remaining zones, monthly TB notification rates ranged from 3.9 to 39.4 per 100 000 population. The five zones with the highest notification rates constituted 62% (95% *CI*: 47–75%) of TB cases and 22% of the population–significantly higher than would be expected if population size and random chance were the only determinants of zone-to-zone variation (48%, 95% simulation interval: 40–59%). These five high-risk zones accounted for 42% (95% *CI*: 34–51%) of the 128 cases detected during the subsequent community-based case finding intervention, which was significantly higher than the 22% expected by chance (*P* < 0.001) but lower than the 62% of cases notified from those zones during the pre-intervention period (*P* = 0.02).

**Conclusions:**

There is substantial heterogeneity in routine TB notification rates at the zone level. Using facility-based TB notification rates to prioritize high-yield areas for active case finding could double the yield of such case-finding interventions.

## Background

More than 10 million people were diagnosed with tuberculosis (TB) in 2018. This burden is not distributed equally; the majority of TB cases are found in 30 countries designated as high burden by the World Health Organization [1]. Even within high-burden countries, TB is geographically heterogeneous, often concentrated in densely-populated, low-income areas [2]. This small-scale geographic heterogeneity, as seen among city neighborhoods, may reflect local transmission [3–5] and is often associated with neighborhood characteristics such as crowding or poverty [6, 7]. Models have suggested that interventions targeted at hotspots could have a large impact on overall incidence [8, 9]. However, in order to be actionable, hotspots would need to be identifiable based on routine data and reasonably stable over the time between hotspot identification and subsequent intervention. Understanding whether these criteria are met could inform local-level prioritization of interventions, as is critical for TB control at the global level [10].

In most high-burden settings, routine TB diagnosis depends on symptomatic presentation by patients, which places the burden on the patient to recognize their symptoms as warranting medical attention and to subsequently seek care. Such symptom-driven diagnosis often fails to detect TB in people with milder symptoms, groups with limited access to care, or areas with limited clinical resources [11, 12]. A recent prevalence survey in Uganda estimated that these current practices fail to detect 46% of TB cases [13]. Active case finding, in which resources are leveraged at the community level to identify TB cases and link them to care, is therefore essential to detect undiagnosed TB in communities [14, 15] and further reduce the burden of TB [16]. However, active case finding is resource intensive, and studies of broad community-wide active case finding have had mixed results [17–21]. Targeted approaches to active case-finding, focusing on people at higher-than-average risk for TB such as recent contacts of TB cases or persons living with HIV, are therefore important [22, 23]. Geographic targeting is an approach to TB case finding that may be feasible but has not been widely implemented, largely because of uncertainty regarding whether cases identified through routine systems can predict the locations of undiagnosed prevalent cases in the community.

A better understanding of local geographic heterogeneity in routinely identified TB cases and the correlation of that heterogeneity with the location of undiagnosed prevalent cases may therefore be useful in directing active case finding interventions to high-risk areas. We used routinely collected TB diagnosis data to identify small-scale geographic areas with high notification rates in Kampala, Uganda. We then evaluated the degree to which these areas contain a higher proportion of undiagnosed prevalent TB, using a subsequent community-wide active case finding intervention.

## Methods

### Study overview and population

This was a community-based study conducted in Kisugu, Wabigalo, and Bukasa parishes in Kampala, Uganda (an area of 2.2 km^2^ with an estimated population of 49 527) from May 2018 through December 2019. The study site consists of 37 contiguous zones; zones are the smallest standard administrative area unit used by the Uganda Bureau of Statistics, with a median size of 0.05 km^2^ within the study area. Prior to initiation of the study, a door-to-door census was conducted by the study team to estimate the population of each zone. Zones with a population of less than 500 were merged with neighboring zones with similar characteristics such that all areas for analysis had a population of at least 500 in order to ensure that each unit of analysis would contain at least two TB cases assuming spatial homogeneity and an anticipated TB prevalence of 400 cases per 100 000 population. Two zones for which the census could not be completed were excluded, resulting in 33 areas for analysis.

### Case definition

A TB case was defined as any individual with a positive sputum smear or GeneXpert result, sputum culture positive for *Mycobacterium tuberculosis*, or documented initiation of TB treatment based on clinical judgment of pulmonary tuberculosis. The GeneXpert (“Xpert”) system (Cepheid, Inc., Sunnyvale, CA, USA) was the primary test used for the study. Sputum samples were tested using Xpert MTB/RIF cartridges at the beginning of the study; the Xpert Ultra cartridge was implemented in February 2019. Sputum smears were used based on clinician request and were rare. Sputum culture was generally only performed for research purposes after TB diagnosis by other means; thus, TB diagnosis based only on culture was very uncommon. In this analysis, we included only individuals who were age 15 years or older and residing within the study area; zone of residence was self-reported and verified using landmarks and Google Maps. We conducted a sensitivity analysis using a case definition that only included microbiologically confirmed (Xpert, smear, or culture) cases.

### Case detection and enrollment

The study prospectively enrolled TB patients in two phases: a facility-based phase (May 2018–January 2019) and a community-based phase (February–December 2019). In the facility-based phase, we enrolled all consenting adult TB cases who lived in the study area and were passively identified through routine TB diagnostic services at four outpatient TB Diagnosis and Treatment Units located within the study area. Clinicians at the facilities were responsible for making TB diagnoses based on clinical judgment and the results of any laboratory tests (for example, sputum smears); diagnosed cases were then referred for study enrollment.

In the community-based phase, we attempted to identify all prevalent TB cases in the community through a combination of passive and active case finding activities. Passive case detection continued at the four health facilities as described above. We also conducted door-to-door sputum collection and testing throughout the study area; this included participants who were at a residence other than their own at the time of testing as long as their residence was within the study area. Ten venue-based screening events were held at churches, markets, and other community locations in order to reach those who were not available during door-to-door testing. Contact investigation was also completed for all identified cases. If residents could be contacted but were not available at the time of screening, follow-up home appointments were scheduled. The goal of the community-based phase was to obtain a sputum specimen from every adult residing in the study area regardless of their TB symptomology.

### Facility-based TB rates

Average monthly TB notification rates (per 100 000 population) for the facility-based study phase were calculated by zone as: (number of TB cases residing in that zone)/(estimated population of the zone×facility-based phase duration, in months). We then ranked zones according to their average monthly TB notification rates and defined a “high-risk” group of zones by starting with the zone reporting the highest TB notification rate and including additional zones with the next-highest rates until the high-risk category accounted for at least 20% of the population. The 20% cutoff was an a priori threshold corresponding to the likely size of any targeted case-finding intervention that could be undertaken in the study area; sensitivity analyses were conducted using cutoffs of 10%, 15%, 25%, and 30% of the population. We calculated the proportion of facility-based phase TB cases who resided within the high-risk group of zones and a corresponding 95% confidence interval (*CI*), assuming a binomial distribution. We compared demographic, clinical, and behavioral risk factors among cases residing in the high-risk vs low-risk zones using Fisher’s exact tests for categorical variables and non-parametric Wilcoxon rank-sum tests for continuous variables.

### Estimation of expected spatial distribution of TB cases

To estimate the number of facility-based TB cases that would be expected to occur in the high-risk zones based on chance alone, we conducted 1000 stochastic simulations in which we assumed that the only driver of spatial heterogeneity in TB notification rates was random variation. For each simulation, we randomly assigned to each zone a number of TB notifications based on population size by drawing a value from a Poisson distribution with mean of (total number of TB cases in study area during facility-based phase×proportion of total population residing in that zone). As with the observed data above, we then sorted the zones by the simulated TB rate (simulated number of TB notifications per 100 000 population per month) and identified the “high-risk” zones as those representing the 20% of the simulated study population with the highest simulated TB notification rates. These simulated high-risk zones therefore occurred randomly throughout the study area, varying from one simulation to the next, and did not correlate with the actual observed high-risk zones. For each simulation, we then calculated the cumulative proportion of TB notifications occurring among residents of these simulated high-risk zones – thereby providing an estimate of the proportion of TB notifications that would be expected to occur in high-risk zones if the only determinant of “high-risk” were random variation in the spatial distribution of TB notifications. We used the 2.5 and 97.5 percentiles of our simulations to define the corresponding 95% uncertainty range around this proportion.

### Stability of facility-based notifications over time

We compared cases diagnosed passively at the health facilities during the facility-based and community-based phases to determine whether there were changes in the spatial distribution of facility-diagnosed cases over time. We calculated the proportion of passively-diagnosed community-phase cases residing in the previously identified high-risk zones with 95% *CI* using a binomial distribution and compared this proportion to the proportion from the facility-based phase residing in those zones using chi-square test.

### Prediction of community-based prevalence using facility-based notifications

We used all cases from the community-based phase to represent the true underlying distribution of prevalent TB. For each zone, we used data from the facility-based phase to calculate an expected number of TB cases that would be found in that zone during the community-based phase by multiplying the proportion of facility-based phase TB cases residing in each zone by the total number of TB cases found in the community phase. The expected number of community-based phase TB cases in each zone was compared to the observed number of TB cases found using chi-squared test. The observed proportion of community-based phase TB cases residing within the high-risk zones (as defined during the facility-based phase) was calculated, with corresponding 95% confidence intervals using a binomial distribution, and compared to the proportion from the facility-based phase using a chi-square test. We also conducted a sensitivity analysis using only community-phase cases that were diagnosed via community-based active case finding (excluding those diagnosed at the health facilities during the community-based phase) to represent the cases that would be expected to be found via a case finding intervention informed by notification data from the facility-based phase.

### Data analysis

All analyses were conducted using Stata 16 (StataCorp, College Station, TX) and maps were created using ArcMap 10.6 (ESRI, Redlands, CA). Categorical variables were presented in percentages and analyzed using Fisher’s exact tests. Continuous variables were presented as median (interquartile range [IQR]) and analyzed using and non-parametric Wilcoxon rank-sum tests. For all comparisons we considered *P* < 0.05 as statistically significant.

## Results

### Facility-based TB notifications

**Fig. 1:**
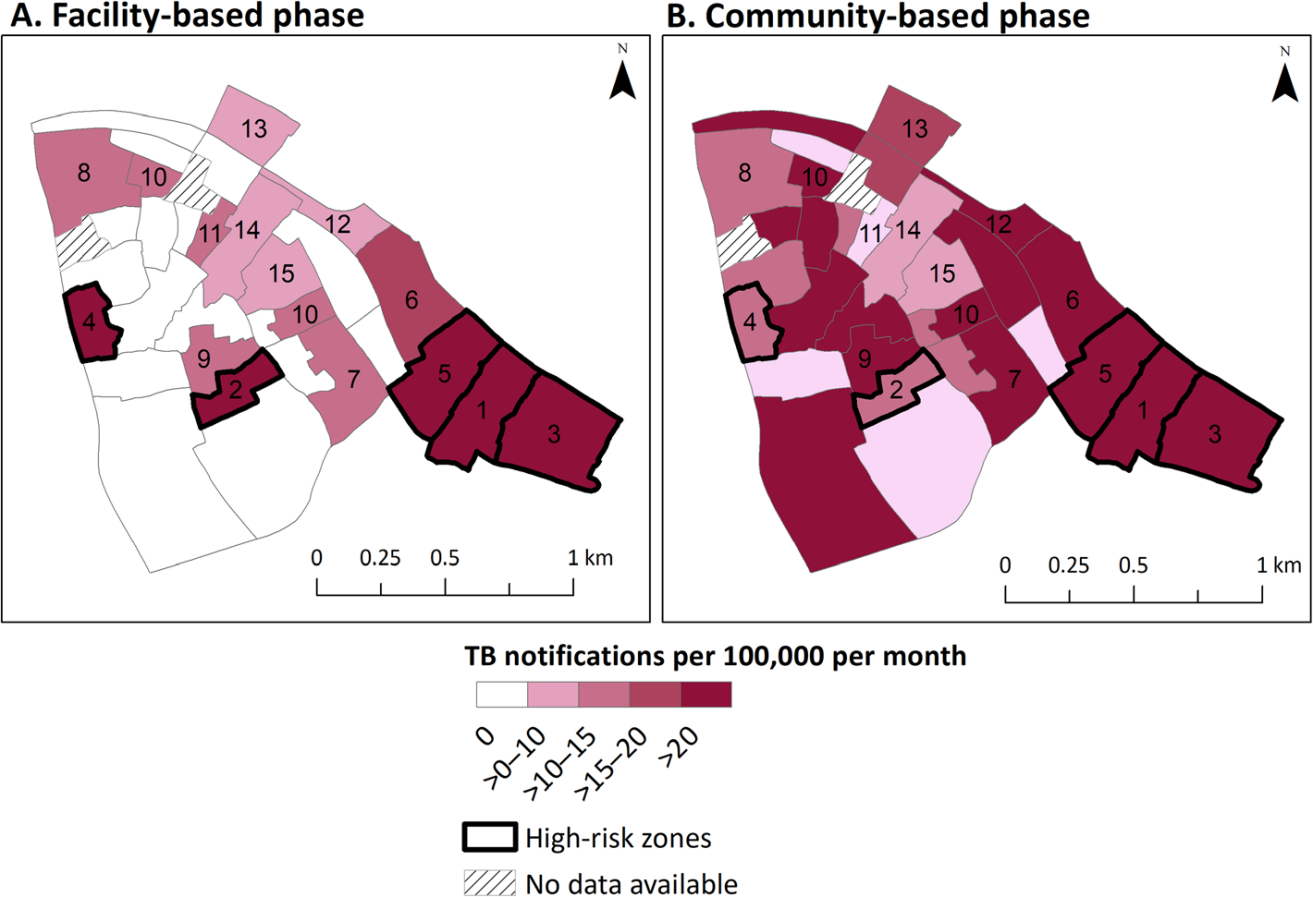
Average monthly tuberculosis notifications, by zone (per 100 000 population). This figure shows the average monthly tuberculosis (TB) notification rate per 100 000 population by zone as estimated in (**a**) the facility-based phase, where TB cases were passively diagnosed via routine standard of care practices from May 2018 to January 2019 and (**b**) the community-based phase, where additional active case finding activities were implemented throughout the study area from February to December 2019. Numbers indicate each zone’s rank (from 1 to 15) based on average monthly TB notification rates during the facility-based phase – with no numbers assigned to zones in which no TB cases were diagnosed during that phase. High-risk zones (outlined in bold) were selected using notifications from the facility-based phase by starting with the zone reporting the highest TB notification rate and including additional zones with the next-highest rates until the “high-risk” category accounted for at least 20% of the population, resulting in five zones. Two zones did not have population data available to inform denominators and were thus excluded from this analysis

During the facility-based phase, 45 cases were notified at the four participating facilities through routine care. These cases resided in 15 different zones in the study area; among those zones, the average monthly TB notification rate ranged from 3.9 to 39.4 TB cases per 100 000 population per month (Fig. 1, panel A). One zone in Bukasa parish accounted for 11 of the 45 (24%) TB cases diagnosed during this phase (Table 1). The five zones with the highest TB notification rates were classified as “high-risk”. These zones accounted for 22% of the population but 62% (95% *CI*: 47–75%) of routinely diagnosed TB cases during the facility-based phase.

**Table 1:** Observed tuberculosis notifications by zone and phase of case detection in urban Uganda

		Facility-based (routine) phase						Community-based (active) phase		
Parish	Zone	Observed TB cases	Population	Monthly TB notification rate (per 100 000)	Rank (Fig. 1)	Cumulative proportion of population	Cumulative proportion of TB cases	Observed TB cases	Monthly TB notification rate (per 100 000)	Cumulative proportion of TB cases
Bukasa	Namuwongo A	11	3299	39.4	1	0.07	0.24	31	120.0	0.25
Kisugu	South B	3	906	39.1	2	0.08	0.31	1	14.1	0.25
Bukasa	Yoka	7	2793	29.6	3	0.14	0.47	13	59.4	0.36
Wabigalo	Klezia	2	950	24.9	4	0.16	0.51	1	13.4	0.37
Bukasa	Namuwongo B	5	2705	21.8	5	0.22	0.62	8	37.8	0.43
Kisugu	Kasanvu	4	2471	19.1	6	0.26	0.71	10	51.7	0.50
Kisugu	South A & C	1	809	14.6	7	0.28	0.73	4	63.1	0.53
Wabigalo	Project	2	1739	13.6	8	0.32	0.78	2	14.7	0.55
Kisugu	Upper Zone	2	1742	13.6	9	0.35	0.82	4	29.3	0.58
Wabigalo	Central	2	1898	12.4	10	0.39	0.87	11	74.0	0.66
Wabigalo	Kitooro	1	1166	10.1	11	0.41	0.89	0	0.0	0.66
Kisugu	Go Down	1	1202	9.8	12	0.44	0.91	7	74.3	0.72
Wabigalo	Industrial	1	1302	9.1	13	0.46	0.93	2	19.6	0.73
Kisugu	Lakeside	2	2701	8.7	14	0.52	0.98	2	9.5	0.75
Kisugu	Mugalasi	1	3062	3.9	15	0.58	1	2	8.3	0.77
18 zones reporting 0 cases in the facility-based phase		0	20 782	0		1.0	1.0	30	18.4	1.0

Compared to facility-based cases from other zones, facility-based TB cases from the high-risk zones were more likely to be female (11/28 [39%] vs 3/17 [18%]), self-employed (10/28 [36%] vs 2/12 [12%]), lower income (median monthly income 340 000 Ugandan Shillings [UGX] vs 600 000 UGX), and HIV positive (11/38 [39%] vs 2/12 [12%]) (Table 2). They were less likely to be able to read and write without difficulty (13/28 [46%] vs 5/17 [71%]) or to have known any other TB cases (7/28 [25%] vs 8/17 [47%]). None of these results was statistically significant due to the small sample size.

**Table 2:** Demographic and clinical comparison between routinely diagnosed cases residing in high risk and low risk zones during the facility-based phase

	Residents of high-risk zones (***n*** = 28)	Residents of low-risk zones (***n*** = 17)	***P***-value
	***n*** (%)	***n*** (%)	
**Female**	11 (39%)	3 (18%)	0.19
**Age at tuberculosis (TB) diagnosis**			0.46
15–24 years	4 (14%)	3 (18%)	
25–34 years	10 (36%)	8 (47%)	
35–44 years	11 (39%)	3 (18%)	
45–54 years	3 (11%)	3 (18%)	
**Literacy**			0.28
Can read & write without difficulty	13 (46%)	12 (71%)	
Can read & write, but one or both are difficult	13 (46%)	5 (29%)	
Can neither read nor write	2 (7%)	0 (0%)	
**Occupation**			0.52
Self-employed	10 (36%)	2 (12%)	
Student	1 (4%)	1 (6%)	
Salaried worker	7 (25%)	6 (35%)	
Occasional work (piece jobs)	4 (14%)	4 (24%)	
Unemployed but able to work	3 (11%)	3 (18%)	
Unemployed and unable to work	3 (11%)	1 (6%)	
**Monthly income (Ugandan Shillings ×1000)**, median (IQR)	340 (135, 600)	600 (350, 750)	0.06
**Skipped 1+ Meals in the last month**^**1**^	19 (68%)	7 (41%)	0.12
**Household Size**, median (IQR)	2 (1, 3)	3 (1, 5)	0.35
**Duration of cough (weeks)**, median (IQR)	5 (3, 12)	8 (4, 20)	0.08
**HIV Positive**	11 (39%)	2 (12%)	0.09
**Ever lived with a TB Case**	6 (21%)	5 (29%)	0.37
**Ever known a TB Case**	7 (25%)	8 (47%)	0.08

^1^ Participant or other adults in their household reported skipping at least one meal or eating smaller meals than wanted because there wasn’t enough money for food

### Expected spatial distribution of TB cases

**Fig. 2:**
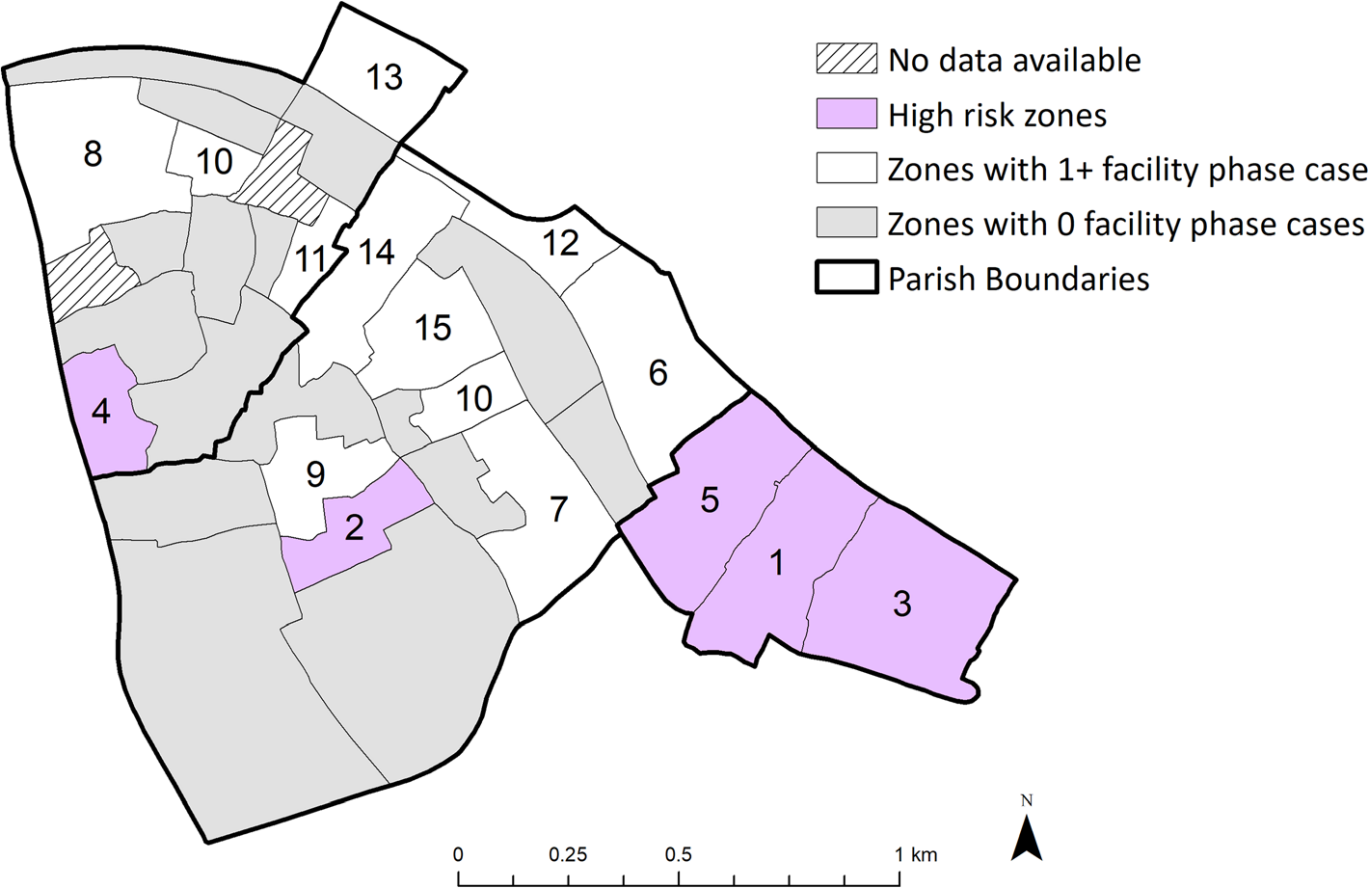
Comparison of observed tuberculosis notifications in high-risk zones to expected cases due to chance. Panel **a** orders the 33 zones the study area according to each zone’s facility-based phase tuberculosis (TB) notification rate (also provided in Table 1); the red line shows the cumulative proportion of TB cases notified who reside in “high-risk” zones (y-axis) according to the cumulative proportion of the population in the high-risk zone (x-axis). The shaded area corresponds to the 95% simulation interval (2.5th and 97.5th percentiles) from 1000 simulations that assume the observed population size in each zone and observed total number of TB notifications, but assign TB cases to zones under the assumption that spatial heterogeneity of TB notifications in the area is driven only by population size and random chance. The vertical line at 22% of the cumulative population represents the cutoff for “high-risk” zones used in our primary analysis and shows that 62% of facility-based cases resided in “high-risk” zones, significantly higher than the corresponding simulation interval of 40–59%. Panel **b** compares the same observed facility-based phase cases from Panel **a** (red line) with the cumulative proportion of TB cases identified through active case finding during the community-based validation phase (blue line), with the zones ordered according to TB notification rates during the facility-based phase. The vertical line in this panel shows that 42% of community-based phase cases resided in the “high-risk” zones (22% of the population) identified based on notifications during the facility-based phase

Under the assumption that the only variation in spatial distribution of TB cases was random chance, we estimated that 47% (95% simulation interval: 39–58%) of TB cases would come from “high-risk” zones accounting for the same fraction of the population (22%), a lower percentage than the observed 62% (Fig. 2, panel A). The results of sensitivity analyses using cutoffs of 10, 15, 25, and 30% of the population are shown in Table 3.

**Table 3:** Sensitivity analysis: Different cutoffs for “high-risk” tuberculosis population

Cutoff for percentage of population in high-risk area	Actual percentage of population in high-risk area^**1**^	Number of zones in the high-risk area	Observed percentage of TB cases in the high-risk area (95% ***CI***)	Expected (simulated) percentage of TB cases in the high-risk area (95% simulation interval)
5%	7%	1	24% (14–39%)	19% (44–26%)
10%	14%	3	47% (32–62%)	35% (28–44%)
15%	16%	4	51% (36–66%)	38% (31–48%)
20%	22%	5	62% (47–75%)	47% (39–58%)
25%	27%	6	71% (56–83%)	55% (46–66%)

^1^ The actual percentage is higher than the cutoff percentage because the actual “high-risk” area consists of intact zones, added sequentially to the “high-risk” area until the cutoff is surpassed

### Stability of facility-based notifications over time

Among passively-diagnosed (health facility) cases during the community-based phase, 32% (95% *CI*: 18–50%) were residents of the high-risk zones as defined by the facility-based phase, significantly lower than would be expected if facility-based diagnoses were constant over time (*P* = 0.009).

### Prediction of community-based prevalence using facility-based notifications

During the community-based phase, 128 people were diagnosed with TB; these individuals resided in 27 different zones. Among these 27 zones, the average monthly TB notification rate ranged from 8.3 to 120.0 TB cases per 100 000 population (Fig. 1, panel B). The five zones classified as “high-risk” based on the facility-based phase (22% of the study population) accounted for 42% (95% *CI*: 34–51%) of the TB cases in the community-based phase (Fig. 2, panel B), which was significantly higher than the 22% expected by chance (*P* < 0.001) but lower than the 62% of cases notified from those zones during the pre-intervention period (*P* = 0.02).

**Fig. 3:**
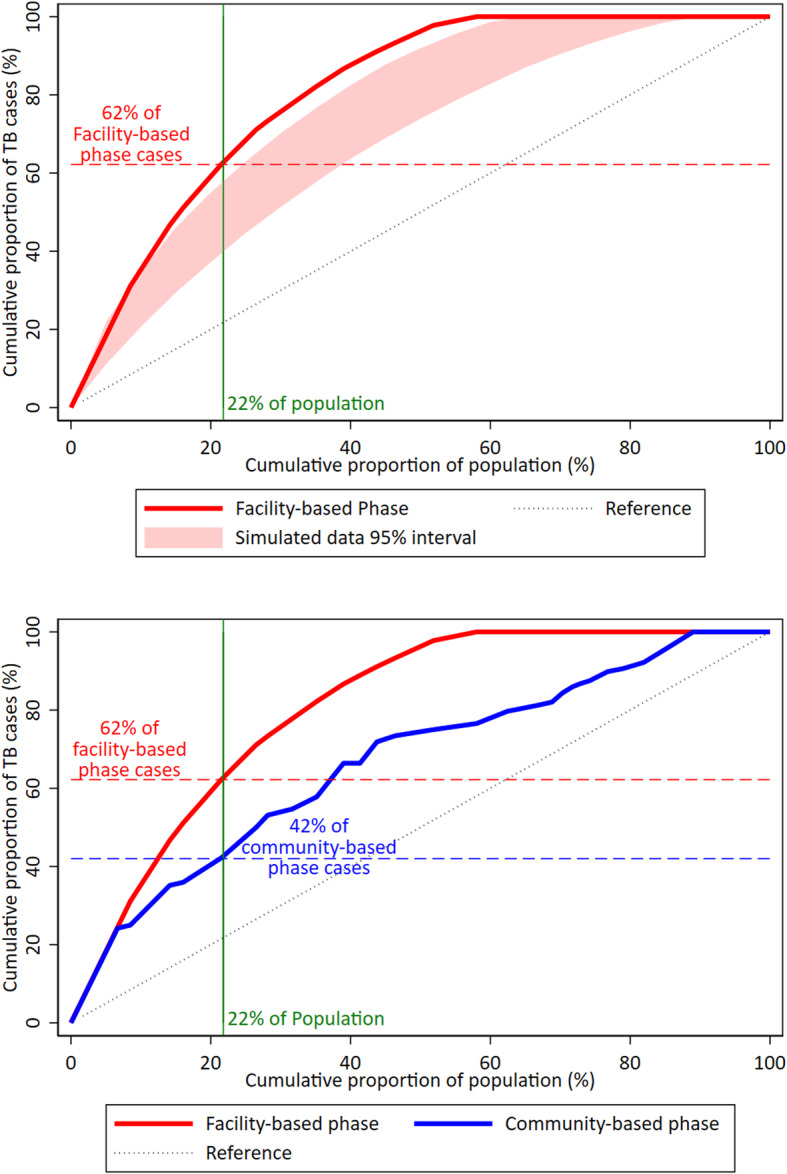
Potential implications of geographic-targeted screening. High-risk zones as defined by the facility-based phase tuberculosis notification rates are indicated in purple. Numbers indicate each zone’s rank (from 1 to 15) based on average monthly TB notification rates during the facility-based phase – with no numbers assigned to zones in which no TB cases were diagnosed during that phase. While targeted active case finding at each selected zone may not be feasible for logistical and political reasons, we highlight that the easternmost three of the five high-risk zones are contiguous and within Bukasa parish (parish boundaries are designated in bold). If this area were to be defined as a priority for case finding activities, it would represent 18% of the total population, 23/45 (51%) of facility-based phase TB cases, and 52/128 (40%) of the community phase TB cases. Two zones did not have population available and were excluded from this analysis

The location of the five high-risk zones is shown in Fig. 3. Three of the five form a contiguous area in Bukasa parish. If this area were to be defined as a single intervention zone, this area would account for 18% of the total population, 51% (95% *CI*: 36–66%) of the routinely diagnosed TB cases in the facility-based phase and 40% (95% *CI*: 32–49%) of TB cases diagnosed in the community-based phase.

### Sensitivity analyses of case definition

When considering only microbiologically confirmed cases (32/45 facility-based phase cases and 125/128 community-based phase cases), six zones accounting for 21% of the population had 59% (95% *CI*: 41–75%) of facility-based phase TB cases; three of these zones were the same as in the primary analysis. We estimated that 53% (95% simulation interval: 43–66%) of TB cases would come from high-risk zones accounting for the same fraction of the population (21%), based on random variation and population size alone. In the community-based phase, 40% (95% *CI*: 32–49%) of cases came from these six “high-risk” zones.

### Sensitivity analyses for active case finding

In the community-based phase, 34 (27%) cases were diagnosed at one of the four health facilities via routine services. In our sensitivity analysis excluding these cases, the five high-risk zones from the facility-based phase in the primary analysis accounted for 46% (95% *CI*: 36–56%) of cases detected via active case finding activities (door to door testing, venue based screening events, and contact investigation).

## Discussion

This study in Kampala, Uganda, found evidence of spatial heterogeneity of TB burden within an urban, densely-populated area using routinely collected TB notification data, with 22% of the population accounting for 62% of cumulative TB notifications. Data from a subsequent community-based active case finding activity demonstrated that routine TB notifications can be used to identify geographic areas with a high underlying burden of TB; the same 22% of the population accounted for 42% of the cases diagnosed during a subsequent case-finding intervention. Geographic targeting could therefore double the yield of active case finding interventions in this setting.

Interventions targeted at small geographical scales have not been widely implemented for TB, but locally focused prevention and case finding interventions have been shown to reduce the burden and transmission of HIV [24], malaria [25], and other neglected tropical diseases [26]. Based on our results, targeting 22% of the population in an urban high-burden area could identify 42% of TB cases in that population. While we chose a cutoff of 20% of the population as a reasonable size to screen, targeted interventions even in this subpopulation would be resource intensive and logistically challenging. To further improve the feasibility of geographically targeted interventions, it may make sense to focus on a single contiguous area. In this study, three of the five “high-risk” zones (Fig. 2) were geographically contiguous, suggesting a possible intervention area. However, this analysis does not account for the increased cost and human resources required to conduct comprehensive interventions in targeted (often underserved) areas with populations that may be highly mobile; in other studies, the per-case-detected costs of active case finding in high TB burden areas have been shown to be high [27, 28]. Intervention-specific cost and epidemiological data would be needed to estimate the impact and cost-effectiveness of any particular intervention in this setting.

Spatial analyses of TB have been primarily limited to using TB notification data [29] and are therefore unable to assess whether high notification rates are due to high prevalence of TB in the community or improved access to TB diagnosis [30]. Numerous studies in high-burden countries have shown that TB notifications are limited by underdiagnosis and under-reporting [14, 15, 31–35], but it is not clear whether the location of residence of the reported TB cases represents that of the missed cases. Our analysis suggests that, in this setting, facility-based TB notifications can reasonably predict the location of prevalent TB cases, suggesting that geographically targeted active case finding using routine notifications to define the target zones could be effective in this area. This is a strength of small-scale geographic analysis in our 2.2 km^2^ study area, as access to health care may be relatively homogeneous. In settings where low notification rates may represent poor access to services, notifications are likely to be less useful in targeting areas for further TB-related interventions.

The population denominators on which our estimates of zone-level TB rates are based used census estimates collected by our research team; official population estimates are not available from the Uganda Bureau of Statistics at this scale, which may limit the ability of other regions to apply these methods. While our population estimates may be imprecise, they are the first to be estimated at the zone level in this area, and there is no a priori reason to expect that any biases in population estimates would be differential from one zone to the next. Our community-based phase was conducted shortly after the facility-based phase, reflecting how a geographically targeted case finding intervention may be implemented, but the lack of stability in geographic distribution of facility-based notifications over time may make it difficult to accurately predict the location of undiagnosed cases. Our sample size was small, leading to imprecise estimates – but such sample sizes are likely to be representative of real-world interventions that might seek to target TB activities on small geographic scales over realistic time frames. Nevertheless, this small sample size results in relatively wide confidence intervals, may affect generalizability, and limits our ability to observe statistically significant differences when comparing residents of high-risk and low-risk zones or facility phase to community phase cases. Finally, given the urban, densely population nature of our study setting, these results may not be generalizable to rural settings or different epidemiological contexts; however, these methods could be applied in different settings using routinely available data.

## Conclusions

This study show that there is substantial geographic heterogeneity in the residence of routinely diagnosed TB patients. We identified high risk zones using data routinely collected at health facilities and show that it may be possible to detect more than 40% of undiagnosed TB cases in the community by screening approximately 20% of the population. Comparison of the spatial distribution of passively diagnosed cases with those identified via community-wide active case finding suggests that geographically prioritized case finding may be an efficient way to detect prevalent TB in urban high-burden settings.

## Data Availability

The data dictionary and code used for this analysis is available on the Johns Hopkins University Data Archive (https://archive.data.jhu.edu/). Data may be made available upon reasonable request.
